# Adaptive Laboratory Evolution of Flavin Functionality Identifies Dihydrolipoyl Dehydrogenase as One of the Critical Points for the Activity of 7,8-Didemethyl-Riboflavin as a Surrogate for Riboflavin in *Escherichia coli*

**DOI:** 10.3390/molecules29245891

**Published:** 2024-12-13

**Authors:** Farshad La-Rostami, Alexandra Scharf, Chenyang Albert, Nils Wax, Marina Creydt, Boris Illarionov, Adelbert Bacher, Stefan Weber, Markus Fischer

**Affiliations:** 1 Hamburg School of Food Science, Institute of Food Chemistry, University of Hamburg, Grindelallee 117, 20146 Hamburg, Germany; fro@wga-hh.de (F.L.-R.); alexandra.scharf@dla-lvu.de (A.S.); chenyang.albert@gmail.com (C.A.); nils.wax@uni-hamburg.de (N.W.); marina.creydt@uni-hamburg.de (M.C.); boris.illarionov@uni-hamburg.de (B.I.); 2 TUM School of Natural Sciences, Technical University of Munich, Boltzmannstraße 10, 85748 Garching, Germany; adelbert.bacher@t-online.de; 3 Institute of Physical Chemistry, Albert-Ludwigs-Universität Freiburg, Albertstraße 21, 79104 Freiburg, Germany; stefan.weber@physchem.uni-freiburg.de

**Keywords:** riboflavin analogs, isoalloxazine, flavoproteins, adaptive laboratory evolution, whole genome sequencing

## Abstract

Riboflavin analogs lacking one methyl group (7α or 8α) can still serve as a surrogate for riboflavin in riboflavin-deficient microorganisms or animals. The absence of both methyl groups at once completely abolishes this substitution capability. To elucidate the molecular mechanisms behind this phenomenon, we performed an adaptive laboratory evolution experiment (in triplicate) on an *E. coli* strain auxotrophic for riboflavin. As a result, the riboflavin requirement of the *E. coli* strain was reduced ~10-fold in the presence of 7,8-didemethyl-riboflavin. The whole genome sequencing of *E. coli* strains isolated from three experiments revealed two mutation hotspots: *lpd*A coding for the flavoenzyme dihydrolipoyl dehydrogenase (LpdA), and *omp*F coding for the major outer membrane protein. In order to investigate the essentiality of flavin’s methyl groups to LpdA, the wild type and mutant variants of *lpd*A were cloned. At least two *lpd*A mutants increased the fitness of *E. coli*, and when 7,8-didemethyl-flavin was added to the growth medium, the increase was significant. To the best of our knowledge, an adaptive laboratory evolution experiment running in triplicate as a tool for the identification of mutation hotspots in the genome of microorganisms exposed to metabolic stress challenges is described here for the first time.

## 1. Introduction

Flavins, mostly in the form of flavin mononucleotide (FMN) and flavin adenine dinucleotide (FAD), are ubiquitous compounds that can mediate one-electron and two-electron transfer in redox transformations. The types of reactions catalyzed by flavoenzymes include dehydrogenation, hydroxylation, dehalogenation, DNA repair, disulfide reduction, and luminescence [1]. For the latter case, light is assumed to serve as the communication between deep-water organisms. Moreover, flavin cofactors are involved in the modulation of protein function by signal transfer. The flavin redox state mediates flavoprotein interactions with other macromolecules such as other proteins, membranes, and nucleic acids. Dynamics in macromolecular interactions initiated by the change in flavin redox state regulate various cellular processes such as circadian rhythm, phototropism, transcriptional regulation, signal transduction, and cell motility [2]. FMN is also a primary regulatory ligand that binds the riboswitch of the riboflavin operon, modulating riboflavin synthesis and transport [3].

**Figure 1:**
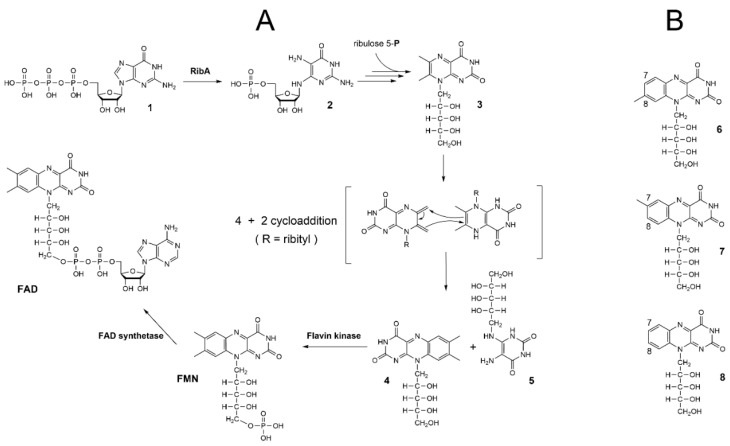
(**A**): A brief scheme of the biosynthesis of flavins from guanosine triphosphate (GTP, **1**) and ribulose 5-phosphate; **2**, 2,5-Diamino-6-(ribosylamino)-4(3*H*)-pyrimidinone 5′-phosphate; **3**, 6,7-dimethyl-8D-ribityllumazine; **4**, riboflavin; **5**, 5-amino-6-ribitylamino-2,4(1*H*,3*H*)pyrimidinedione; FMN, flavin mononucleotide; FAD, flavin adenine dinucleotide. RibA, GTP cyclohydrolase II. (**B**): **6**, 7-demethyl-riboflavin; **7**, 8-demethyl-riboflavin; **8**, 7,8-didemethyl-riboflavin.

Riboflavin—the biosynthetic precursor of FMN and FAD—is synthesized from guanosine triphosphate (GTP) and ribulose 5-phosphate (Figure 1A). It is assumed that the electron transfer ability of flavins is strongly associated with the electron signature of the isoalloxazine ring system [4]. For a long time, two methyl groups (7α and 8α) at the isoalloxazine motif were considered only as remnants of the riboflavin synthase reaction, where they play an indispensable role in mediating the 4 + 2 cycloaddition between two molecules of 6,7-dimethyl-8-ribityllumazine (Figure 1A) [5]. Recently, it was shown that the presence or absence of those methyl groups (one or both of them) influences the photophysical properties of FMN derivatives in the absence and presence of a protein environment [6]. Yet, important characteristics such as the capability to accept or donate electrons (reduction-oxidation potential) were changed only slightly.

Since the chemical structure of riboflavin was determined in the 1930s, different riboflavin analogs have been synthesized, some of which were tested as riboflavin surrogates on weanling rats and newly hatched chicks, which were eventually devoid of riboflavin-producing gut microbiota, and on the riboflavin-dependent bacterium *Lactobacillus casei* [7]. Compounds with different substitutions at positions 7 and 8 of the isoalloxazine ring system were among those riboflavin structural analogs. It has been shown that ethyl, chlorine, bromine, or hydrogen substitutions for any of these methyl groups did not abolish the capability of the resulting derivative to restore the growth of *L. casei*, although most of them did not afford complete restoration in comparison to riboflavin [8]. In contrast to this, the riboflavin derivative with either chlorine or hydrogen as substitutes for methyl groups simultaneously at both positions 7 and 8 of the isoalloxazine motif lost the riboflavin surrogate ability for *L. casei* completely. The latter seems a baffling finding because the hydrogen substitution for a methyl group represents the least possible scaffold modification in the flavin structure. From the mechanistic point of view, the binding of such a derivative by flavoproteins should not be structurally hindered because the absence of methyl group(s) makes the isoalloxazine motif a bit smaller and, as far as we know, these groups do not participate in charge-, polar- or hydrogen-bond interactions. The structures of mono- and didemethyl-riboflavins are shown in Figure 1B.

In this study, we show that for the model organism *Escherichia coli*, both monodemethyl-riboflavins (**6** and **7**) can serve as surrogates for riboflavin, whereas the 7,8-didemethyl-riboflavin (**8**) cannot. Therefore, there must be constituents of the *E. coli* flavoproteome and/or of the regulatory network whose function abolishes when methyl groups on the isoalloxazine motif are absent. In order to identify those constituents, we performed an adaptive laboratory evolution experiment on the riboflavin auxotrophic strain of *E. coli* with a limiting riboflavin concentration and excess of **8** in the growth medium. As an intermediate result, within a time period of ~10^3^ cell divisions, the limiting riboflavin concentration in the growth medium could be diminished by one order of magnitude. Whole genome sequencing revealed that in three strains adapted to the low riboflavin concentration in three independent evolution experiments, 29% of generated mutations were located only in two genes: *lpd*A coding for the flavoenzyme dihydrolipoyl dehydrogenase and *omp*F coding for the outer membrane protein. The *lpd*A variants were cloned and investigated regarding their ability to enhance the growth of the riboflavin deficient strain in the presence of **8**.

## 2. Results

### 2.1. Preparation of E. coli BL21(DE3)-ΔribA Strain

In order to construct the *E. coli* BL21(DE3) strain auxotrophic for riboflavin, the *rib*A gene coding for GTP-cyclohydrolase II that catalyzes the first committed reaction of the pathway (see Figure 1A) was chosen as a target for genome editing using the clustered regularly interspaced short palindromic repeats (CRISPR) technique [9]. The target DNA sequence (also referred to as the protospacer region) for Cas9 nuclease within the *rib*A gene was selected using the online CRISPR-ERA tool [10]. The detailed procedure for the CRISPR-Cas9 mediated inactivation of the *rib*A is described in the “Supplementary Materials”.

### 2.2. Testing of Demethyl-Riboflavins as Riboflavin Surrogates for the E. coli BL21(DE3)-ΔribA Strain

The resulting *E. coli* BL21(DE3)-Δ*rib*A strain was able to grow when the growth medium was supplemented with more than 200 mg/L riboflavin (≥0.53 mmol/L). In order to decrease the growth-limiting riboflavin concentration, the strain was transformed with the plasmid pREP-RT (see “Materials and Methods”) that bears a riboflavin transporter gene from *C. glutamicum*. For reasons of simplicity, the resulting *E. coli* strain BL21(DE3)-Δ*rib*A[pREP-RT] is henceforward referred to as Δ*rib*A-RT. The growth-limiting riboflavin concentration for this strain was about 10 nmol/L.

**Figure 2:**
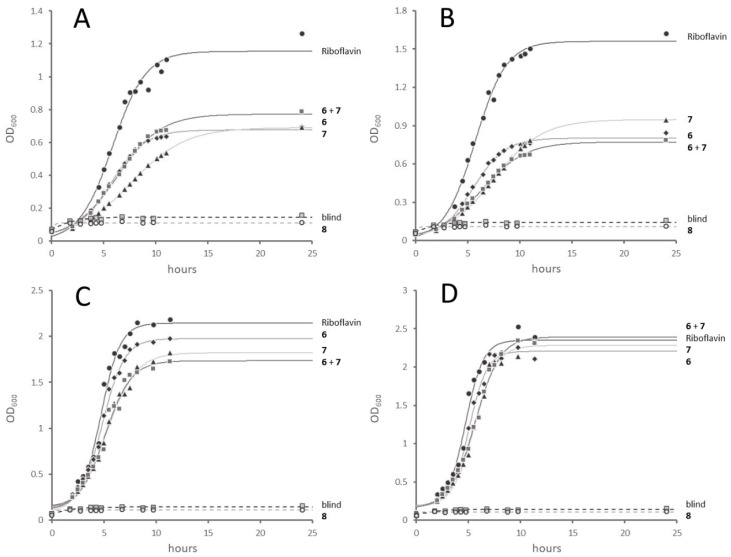
Typical cultivation curves of the ΔribA-RT strain on synthetic M9 medium with riboflavin or riboflavin analogs. For the structures of the riboflavin analogs **6**, **7**, and **8**, see Figure 1B. The growth curves for the cultures containing either riboflavin or **6** or **7** are indicated, respectively. The growth curves marked as **6** + **7** indicate cultivation cultures with both **6** and **7** in equal concentrations. The concentration of flavin species in the growth medium containing either riboflavin or **6** or **7** or both **6** and **7** was 15 nmol/L (**A**), 30 nmol/L (**B**), 50 nmol/L (**C**), 100 nmol/L (**D**). “Blind” indicates the growth curve for the culture with no flavin added. The “**8**” indicates the growth curve for the culture with only **8** added (0.5 µmol/LM, all panels).

All three demethyl-riboflavins (**6**, **7**, **8**) were tested as surrogates for riboflavin in Δ*rib*A-RT, as shown in Figure 2. For this purpose, the Δ*rib*A-RT cells were grown in five separate shaking flasks, each with M9 synthetic medium supplemented with kanamycin (Km, 20 mg/L), as well as with one of the demethyl-riboflavins or riboflavin. In four experiments, the concentrations of riboflavin or monodemethyl-riboflavins were set to 15 nmol/L, 30 nmol/L, 50 nmol/L, or 100 nmol/L. One culture with **8** (0.5 µmol/L) as a potential substitute for riboflavin and one blank culture without any flavin additive were run in parallel. Figure 2 shows that riboflavin analogs **6** or **7** could restore the growth of the Δ*rib*A-RT cells in the absence of riboflavin fully (50 nmol/L or 100 nmol/L) or in part (30 nmol/L or 15 nmol/L) in comparison to riboflavin. In contrast to this finding, **8** could not restore the growth of the Δ*rib*A-RT strain.

### 2.3. 7,8-Didemthyl-riboflavin Is Transported into ΔribA-RT Cells by the Riboflavin Transporter from C. glutamicum

The inability of **8** to restore the growth of the Δ*rib*A-RT strain on the riboflavin-free growth media raised the question of whether **8** could be transported from the growth medium into the *E. coli* cells by the *C. glutamicum* riboflavin transporter. To examine this possibility, the Δ*rib*A-RT strain was transformed with the plasmid pT7-HISLOVC450A that was earlier used for the production of the FMN-binding light-oxygen-voltage-sensing (LOV2) protein [6]. The resulting strain was grown on the synthetic M9 medium supplemented with kanamycin (20 mg/L), ampicillin (50 mg/L), riboflavin (1.9 µmol/L), and **8** (3.0 µmol/L). The expression of the LOV2 gene was started by the addition of isopropyl β-D-1-thiogalactopyranoside (IPTG). The protein was isolated and purified as described in “Materials and Methods”. The flavin ligands isolated from the LOV2 protein were analyzed using a liquid chromatography instrument coupled to a mass spectrometer (LC-MS) (Figure S3). According to these results, the isolated ligand mixture consisted of FMN and 7,8-didemethyl-FMN in a ratio of approximately 1.0 to 1.3. In other words, the **8** is transported into *E. coli* cells and its inability to act as a riboflavin surrogate must have other causes.

### 2.4. Redox Behavior of the Demethyl-Flavins

One reason for the inability of **8** to act as a substitute for riboflavin in riboflavin deficient *E. coli* could be its anomalous redox behavior. Previously published electrochemical data on FMN, as well as phosphorylated **6**, **7**, and **8** [6], have shown that for the phosphorylated **8** as a free ligand, the redox potential (E_1/2_ = −361 mV) was close to that of the FMN (E_1/2_ = −402 mV), suggesting that there would be no serious problems regarding the uptake, release, and transport of electrons, as well as other biochemical events for which the redox potential value of the flavin is important.

### 2.5. Adaptive Evolution Experiment

In order to discover the acting points of methyl groups of flavins in *E. coli*, a laboratory evolution experiment was started with the riboflavin auxotroph strain Δ*rib*A-RT cultivated in the growth medium with limiting concentrations of riboflavin and a much higher concentration of **8**. It was expected that some of the mutations accumulated in the course of this experiment would positively or negatively influence bacterial fitness, whereas the rest would be neutral (no gain, no loss). When a number of genomic mutations were accumulated, it might be difficult to sort the ones beneficial for the bacterial growth from the non-beneficial ones, especially considering that a substantial part (~35%) of the ~4300 open reading frames found in the genome of *E. coli* still remained unannotated [11]. For this reason, not one but three evolution experiments were started in parallel and were run strictly separately. The respective *E. coli* cultures are henceforward referred as evolution lines 1, 2, and 3. When mutations in a certain DNA region, like an open reading frame, positively contribute to the bacterial growth capability in the presence of **8**, the accumulation of mutations in that region in more than one evolution line would become a strong indication of such contribution.

For the starting culture, 1.5 mL of M9 synthetic medium supplemented with Km (20 mg/L), 9 nmol/L to 12 nmol/L riboflavin, and 0.5 µmol/L **8** were inoculated with one fresh colony of Δ*rib*A-RT. The liquid culture was incubated at 32 °C for 24 h in a shaking flask (sterile 15-mL polypropylene tube with a screw cap). For further cultivations, an aliquot of 1.5 µL was transferred from a grown-up culture (referred to as a previous passage) into 1.5 mL of the fresh medium, thereby starting a new culture (referred to as a new passage). Approximately 24 h after re-inoculation, the OD_600_ of the culture was measured. When the OD_600_ value was higher than 1.0, the riboflavin concentration in the growth medium for the next passage was reduced by ~5%. When the OD_600_ was between 0.3 and 1.0, the riboflavin concentration in the next passage medium remained unchanged. When it was lower than 0.3, the riboflavin concentration in the growth medium for the next passage was increased by ~5%.

**Figure 3:**
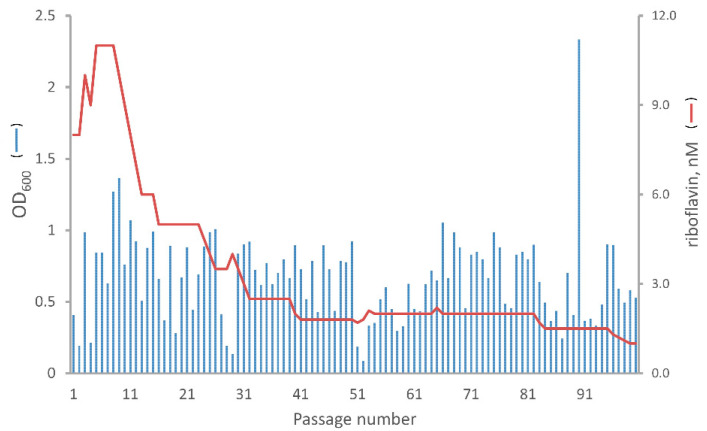
OD_600_ and riboflavin concentration profile of evolution line 1. The blue bars represent the OD_600_ values at the end of the respective passages. The red line represents the riboflavin concentration (nmol/L) in the growth medium at the start of the respective passage.

Figure 3 shows exemplarily the dynamics of absorbency value and riboflavin concentration for one of the three evolution lines spread over 100 passages. The respective data for the two remaining evolution lines are shown in Figure S4.

In the course of the evolution experiment, the growth-limiting riboflavin concentration in each line could be reduced from about 10 nmol/L to approximately 1 nmol/L within 100 cultivation passages.

### 2.6. Genome-Wide Mutation Analysis

Three clones auxotrophic for riboflavin, one from each evolution line, were isolated from the cultivation passage 100. Whole genome sequencing of genomic DNA of those clones plus the Δ*ribA*-RT starting strain was conducted as described in the “Materials and Methods” section. The sequencing reads were mapped to the published genome sequence of the *E. coli* BL21(DE3) strain (accession NC_012892.2). Mapped reads were manually inspected for variants using the Integrative Genomics Viewer (IGV) [12]. Finally, for a genome wide mutation analysis, the resulting genomic DNA sequences of the three clones were compared with that of the Δ*ribA*-RT. For the evolution lines 1, 2, and 3, the number of mutations evolved in the course of the experiment were 7, 5, and 12, respectively. The list of all identified sequence variations is shown in Table S3. Further analysis of these revealed that 7 of the 24 mutations were found in only three genome DNA regions, as shown in Table 1. Except for those three genome regions, no other single genome region (open reading frame or intergenic region) contained more than one mutation.

Three different single mutations (single nucleotide polymorphism or SNPs) in three different evolution lines were identified in the gene *lpd*A coding for dihydrolipoyl dehydrogenase—an FAD-containing flavoprotein that is part of the ketoacid dehydrogenase multienzyme complex and the glycine cleavage system and catalyzes the reoxidation of the lipoamide cofactor bound to a dihydrolipoamide transacetylase [13,14]. Two SNPs and two deletions spread among all evolution lines were found in the *omp*F gene coding for the outer membrane protein, which forms pores that allow passive diffusion of small molecules across the outer membrane [15]. Finally, evolution lines 1 and 2 both revealed the identical SNP (C → T) in the non-coding region between the *aph*A gene coding for acid phosphatase and the open reading frame B21_RS2502. Except for *lpd*A, no other genes for flavoenzymes were identified among the open reading frames that contained new mutations.

### 2.7. Cloning of lpdA

LpdA is potentially of high interest in the question of whether **8** can be used as a substitution for FAD in the enzyme. To investigate this, four gene variants for dihydrolipoyl dehydrogenase (*lpd*A) (one wild type and the three mutant variants shown in Table 1) were amplified in four PCR sets and cloned in pET22b(+) as described in the “Materials and Methods” section. As a source of genomic DNA for PCR, *E. coli* colonies were used from each evolution line at the cultivation passage number 100 and a colony of the Δ*rib*A-RT.

The cloned DNA fragments were sequenced using standard Eurofins Genomics primers pET-RP and T7. The resulting nucleotide sequences of the *lpd*A clones (see Figure S5) were identical to the respective nucleotide sequences determined by next-generation sequencing (NGS). The inferred amino acid sequences are shown in Figure S6.

### 2.8. Influence of the Mutated lpdA Genes on the Fitness of the ΔribA-RT Strain

To confirm exemplarily the effect of the mutations shown in Table 1 on bacterial fitness in the presence of **8**, we chose the *lpd*A gene for conducting several in vivo experiments. The investigation of the sole effect of the *lpd*A mutants on bacterial fitness in the presence or absence of **8** in the growth medium must be done with an identical genetic background. Consequently, four plasmids with cloned *lpd*A variants (WT or three mutants) were transformed into the ∆*rib*A-RT. Since LpdA is a part of the ketoacid dehydrogenase multienzyme complex, overexpression of the *lpd*A variants alone would not have made sense because other subunits of the multienzyme complex would have been present in the cell at much lower concentrations. Since *E. coli* BL21(DE3) shows low but detectable basal expression of target genes [16], we decided to carry out the experiment without the addition of IPTG to the growth medium. Under these conditions, the molar ratio of the native LpdA and mutated LpdA variants in ketoacid dehydrogenase multienzyme complex was not known in advance. Therefore, the experiment was carried out at a riboflavin concentration of 9 nmol/L, near the limiting value for the ∆*rib*A-RT.

Strains obtained after transformation of the Δ*rib*A-RT with the plasmids pET-*lpd*Awt and pET-*lpd*Am1 to pET-*lpd*Am3 (Table S4) were grown in liquid synthetic M9 medium with riboflavin, and for each strain, two cultivation experiments were carried out: with or without 0.5 µmol/L **8** in the growth medium. These sets of experiments were repeated three times; the OD_600_ values were measured at the end of the exponential growth phase of each culture, and for each *lpd*A variant, a quotient of the OD_600_ value of the culture with **8** to the OD_600_ value of the culture without **8** was calculated. The results are summarized in Table 2.

Obviously, at least for the *E. coli* strains 2 and 4 (Table 2), the presence of **8** in the growth medium slightly but significantly accelerated their growth, whereas for the strain with the wild-type *lpd*A, the growth in the presence of **8** was somewhat slower. 

## 3. Discussion

Flavins play many key roles in living organisms. Several natural riboflavin analogs were discovered in the last 80 years. Some of them, like molybdopterin, F420, or prenylated FMN, serve as enzyme cofactors in different groups of organisms [17,18,19]. Other compounds such as roseoflavin are natural riboflavin antagonists with an antibiotic effect [20]. Aside from that, many artificial analogs of riboflavin have been synthesized, and their biological activity has been extensively studied [7,8]. Among such compounds were those with different substituents (hydrogen, ethyl, chlorine, bromine) for methyl groups at C7 and C8 of the isoalloxazine ring. They have been used for the investigation of the flavoproteome in gram-positive bacteria, yeasts, mammals (weanling rats), and birds (chicks) [21,22,23,24]. Interestingly, while some analogs were found to be riboflavin antagonists, others proved to be riboflavin surrogates that could completely replace riboflavin in the diet of riboflavin-dependent organisms. The question of at which specific points in the flavoproteome or in the regulatory network of metabolic fluxes such effects take place remained unclear. Given this background, it is no surprise that the idea to develop a potent and universal antibiotic from one of riboflavin analogs that interferes with one of the flavin functions or riboflavin biosynthesis, like sulfonamides with the folic acid synthesis, has been raised several times [25,26].

The fact that the presence of at least one of the methyl groups is indispensable in fulfilling one or more vitally important functions of flavins in *E. coli* appears enigmatic. In this study, we reported that either 7-demethyl-riboflavin (**6**) or 8-demethyl-riboflavin (**7**) may restore the growth of the riboflavin-deficient *E. coli* strain when added to the synthetic riboflavin-free medium, whereas 7,8-didemethyl-riboflavin (**8**) did not show such an effect. Obviously, there must be one or several flavin action points in the metabolic or regulatory network of *E. coli* where the presence of at least one methyl group on the isoalloxazine motif (either at position C7 or C8) is of crucial importance. In order to identify those flavin action points, we carried out the adaptive evolutionary experiment of cultivating the riboflavin-deficient *E. coli* strain in synthetic medium with an adjustable limiting concentration of riboflavin and a high excess of **8**. The experiment was performed in triplicate. No ionizing radiation or mutagenic chemicals were used to increase the mutagenicity rate. Still, the limiting concentration of riboflavin in the growth medium could be decreased from ~10 nmol/L to ~1 nmol/L within 100 passages. The issue that was considered further was the following: how many mutations are expected to occur within 100 passages? The natural mutation rate for *E. coli* is ~2 × 10^−9^ mutation per base and generation [27]. With the number of cells increasing about 1000 times per cultivation passage (the dilution factor by inoculating an aliquot of the “old” culture into the fresh medium is 1:1000), approximately ten cell generations should pass (1000 ≈ 2^10^). For our evolution experiment, this meant that in *E. coli* with the genome size of ~5 × 10^6^ bp, only about one in ten cells would get one new mutation in the course of one cultivation passage (2 × 10^−9^ × 5 × 10^6^ × 10 = 0.1), or about 10 mutations per cell accumulated within 100 passages. For this reason, we decided to isolate single clones from every evolution line and sequence its genomic DNA. Remarkably, the number of mutations revealed via the analysis of genome sequencing data was in very good agreement with the estimation made above (cf. Table S3). The analysis of genome sequencing data identified genes for dihydrolipoyl dehydrogenase (*lpd*A) and outer membrane protein (*omp*F) as bearing new mutations in all three evolution lines. Both proteins are of high natural abundance in *E. coli* [28,29]. The outer membrane protein mediates the non-specific diffusion of small molecules like sugars, ions, and amino acids with an exclusion limit of ~600 Da; however, the penetration rates are based not only on the size but also on the hydrophobicity and charge of the solute [30]. To what extent the identified mutations in *omp*F may increase the fitness of the riboflavin-deficient strain can only be speculated upon for the time being. For example, it is conceivable that mutations may decrease the possible passive diffusion of riboflavin away from the cell, thereby increasing its intracellular concentration.

The other gene mutated in all evolution lines—*lpd*A—codes for dihydrolipoyl dehydrogenase. This enzyme is a part of the glycine cleavage system, as well as the α-ketoglutarate dehydrogenase and pyruvate dehydrogenase complexes. The latter provides the link between glycolysis and the tricarboxylic acid cycle, enabling the full oxidation and the full energy gain from sugar molecules entering glycolysis. This is one of the reasons why dihydrolipoyl dehydrogenase is one of the most abundant proteins in *E. coli* and may therefore hold a great part of the FAD in the cell in comparison to other flavoproteins. This, in turn, would explain the decrease in the growth-limiting concentration of riboflavin by one order of magnitude when a mutation in this gene enables the respective enzyme variant to acquire the 7,8-didemethyl-FAD as a substitute for FAD.

The surrogate effect of the **8** for the riboflavin was investigated for four riboflavin deficient *E. coli* strains that were genetically identical, except for *lpd*A, which was present either as wild type or as one of the mutant variants identified and isolated in the course of the adaptive evolution experiment. At least in the presence of the two *lpd*A mutant genes, the cells grew faster with added **8** than without it, whereas for the strain with the only wild-type *lpd*A, no such behavior was observed. This fact indicates that the **8** in combination with the *lpd*A mutants increases bacterial fitness under limiting concentrations of riboflavin. The investigation of the mechanisms that produce such an effect follows below.

## 4. Materials and Methods

### 4.1. Chemicals and Enzymes

The water (ρ > 18 MΩ × cm) used for all preparations was purified with Millipore Direct-Q 3UV-R (Merck, Darmstadt, Germany). All chemicals and growth media components were purchased from Carl Roth (Karlsruhe, Germany) if not otherwise mentioned. D/L-arabinose and trichloroacetic acid were purchased from Merck (Darmstadt, Germany). The enzymes (restriction endonucleases, T4 DNA ligase, and Phusion high-fidelity DNA polymerase) were purchased from New England Biolabs (Frankfurt am Main, Germany). Plasmid DNA was isolated from *E. coli* using a Monarch plasmid miniprep kit (New England Biolabs GmbH, Frankfurt am Main, Germany). Amplicons were isolated from agarose gel or from PCR samples using a peqGOLD gel extraction kit (VWR LLC., Erlangen, Germany) or a Monarch PCR and DNA cleanup kit (New England BioLabs, Frankfurt am Main, Germany), respectively. DNA quantitation was carried out using a Quantus™ fluorometer with the associated QuantiFluor dsDNA System (Promega GmbH, Walldorf, Germany) according to the manufacturer’s instructions. Nucleotide sequences of newly constructed plasmids or PCR fragments were determined using the external sequencing service of Eurofins Genomics (Eurofins Genomics Europe Shared Services GmbH, Ebersberg, Germany). The amplification of DNA fragments from genomic or plasmid DNA was performed using Phusion high-fidelity DNA polymerase. Colony PCR was carried out using home-made Taq DNA polymerase [31]. The synthetic *pnu*X gene coding for the riboflavin transporter of *Corynebacterium glutamicum* was purchased from GenScript (Rijswijk, The Netherlands). The synthetic M9 medium contained Na_3_PO_4_ (12 g/L), KH_2_PO_4_ (3 g/L), NaCl (0.5 g/L), MgCl_2_ (0.5 mmol/L), CaCl_2_ (0.25 mmol/L), NH_4_Cl (2.5 g/L), riboflavin-free casein hydrolysate (6 g/L), D-glucose (3 g/L), and trace elements (MnCl_2_, 0.08 mmol/L; CuCl_2_, 0.09 mmol/L; CoCl_2_, 0.11 mmol/L; FeCl_3_, 0.23 mmol/L; Ni citrate, 0.33 mmol/L; Zn acetate, 0.42 mmol/L).

### 4.2. Demethylated Riboflavins

Three riboflavin analogs—7-demethyl-riboflavin (**6**), 8-demethyl-riboflavin (**7**), and 7,8-didemethyl-riboflavin (**8**) (see Figure 1B)—were synthesized as described earlier [32]. The concentration of flavin species in the flavin stock solutions was determined as described in the Supplementary Materials section. Riboflavin and its demethyl variants were purified using the Agilent HPLC System 1100 (Agilent Technologies Deutschland GmbH, Waldbronn, Germany) equipped with an RP18 column (4.6 mm × 250 mm, 5 µm; Macherey-Nagel GmbH & Co. KG, Düren, Germany). The mobile phase used 35% methanol (HPLC grade) in water with 0.1 mol/L ammonium formate under isocratic conditions at the flow rate of 1.0 mL/min. Injection volumes were 50 µL. The eluate was checked for flavins using an Agilent diode array detector (G1315B, wavelength 470 nm) and fluorescence detector (G1321A, excitation 440 nm, emission 530 nm). The retention volumes for the flavins were as follows: riboflavin, 4.9 mL; **6**, 3.7 mL; **7**, 3.8 mL; **8**, 3.2 mL. The eluate fractions containing one and the same flavin analog were combined, evaporated using a rotary evaporator (Heidolph Instruments GmbH & Co. KG, Schwabach, Germany), dissolved in water, sterilized by passing through a sterile 0.2 µm syringe filter (Macherey-Nagel GmbH & Co. KG, Düren, Germany), and stored at −20 °C. The analytical chromatograms of all four flavins are shown in Figure S7.

### 4.3. Bacterial Strains, Plasmids and DNA Primers

The bacterial strains and plasmids used in this study are shown in Table S4. All primers used in this study were purchased from Integrated DNA Technologies, Inc. (Leuven, Belgium) and are depicted in Table S2.

### 4.4. Transformation of E. coli Cells

*E. coli* electrocompetent cells were prepared as described earlier [33]. Electrocompetent cells were transformed either with DNA purified from ligation samples (strain XL1) or with a single plasmid DNA clone (strain BL21(DE3)). For one transformation, an aliquot of 50 µL of the electrocompetent cells together with 1 ng of DNA were transferred into a 1 mm electroporation cuvette (Electroporation Cuvette Plus, BTX^®^ Harvard Apparatus, Holliston, MA, USA). A high voltage pulse (1800 V) was applied using the electroporator ECM 399 (BTX^®^ Harvard Apparatus, Holliston, MA, USA). The cells were transferred from the electroporation cuvette into 1 mL of SOB medium (1% (*w*/*v*) casein hydrolysate, 0.5% (*w*/*v*) yeast extract, 10 mmol/L NaCl, 2.5 mmol/L KCl, 10 mmol/L MgCl_2_, 10 mmol/L MgSO_4_) and incubated for 1 h in a shaking flask (150 rpm, 37 °C). Finally, the cells were harvested using centrifugation (4500× *g*, 7 min, 4 °C) and the cell pellet was resuspended in 0.1 mL of SOB medium and then plated on LB agar containing the appropriate antibiotics.

### 4.5. Colony PCR

For the identification of *E. coli* colonies harboring the desired genetic modifications on the chromosome or plasmid, a modified method of colony PCR was used [15]. One colony from the freshly streaked agar plate was stirred in 50 µL water. An aliquot of 2 µL that served as a DNA template source was transferred into 50 µL of Thermopol PCR buffer (20 mmol/L Tris-HCl pH 8.8, 10 mmol/L (NH_4_)_2_SO_4_, 10 mmol/L KCl, 2 mmol/L MgSO_4_, 0.1% Triton^®^-X-100, 0.2 mmol/L of each dNTP, 0.5 µmol/L of each primer). A total of 0.5 U of home-made Taq DNA polymerase was added to the PCR sample, and PCR was initiated via incubation at 94 °C for five minutes for initial DNA denaturation, followed by 25 to 30 DNA polymerization cycles. The resulting PCR products were analyzed using agarose gel electrophoresis.

### 4.6. Construction of pTargetF-ribA and Synthesis of the Editing Template for the Knockout of ribA

In order to introduce a DNA fragment encoding the target-specific 20 nucleotide-long 5′ end of the sgRNA coding region into the plasmid pTargetF, a site-directed mutagenesis by double inverse PCR [34] was carried out using Phusion high-fidelity DNA polymerase and two pairs of primers as shown in Table S5. DNA was transformed into *E. coli* XL1 electrocompetent cells and clones that carried the resulting plasmid pTargetF-*rib*A were identified with colony PCR using the primers ribAfw2 and ribAr.

The editing template for the knockout of the *rib*A gene was synthesized by amplification using primers AmL1, AmL2, AmR1 and AmR2. Genomic DNA of one colony from the freshly plated *E. coli* BL21(DE3) was used as the template. The detailed procedure is described in the Supporting Information section (Table S6). The nucleotide sequence of the editing template is shown in Table S1.

### 4.7. Transformation of E. coli BL21(DE3) Cells for the Knockout of ribA

Cells were grown at 30 °C in LB medium containing Km (25 mg/L) in a shaking flask (150 rpm) until the OD_600_ = 0.4. A sterile solution of L-arabinose was added to a final concentration of 10 mmol/L for induction of the lambda *red* genes, coding for Exo, Beta, and Gam proteins [35]. The cells were harvested for treatment with 10% glycerol when the OD_600_ reached 0.7.

For electroporation, 50 µL frozen aliquot of the electrocompetent cells was thawed on ice, mixed with editing template DNA (ETribA, 100 ng) and pTarget-*rib*A DNA (60 ng). The electroporation was carried out as described in the section “Transformation of *E. coli* cells”. The cells were transferred from the electroporation cuvette into 2 mL of SOB medium containing 10 mmol/L L-arabinose and 0.08 mmol/L riboflavin and incubated for 2 h in a shaking flask (150 rpm at 30 °C). Cells were harvested using centrifugation (4000× *g*, 7 min, 4 °C) and the cell pellet was resuspended in 0.1 mL of SOB medium and then plated on an LB agar plate containing Km (25 mg/L), spectinomycin (30 mg/L), L-arabinose (1.5 g/L), and riboflavin (200 mg/L). The agar plates were incubated at 30 °C for 18–24 h. From *E. coli* transformants the pTarget-*rib*A plasmid was cured using the method of Jiang et al., 2015 [9]. Thereafter, the pCas plasmid was cured by growing the *E. coli* colonies overnight at 42 °C on LB agar without antibiotics. The following morning, the colonies that appeared on the plate were assessed for plasmid loss by testing their sensitivity to Km (25 mg/L).

### 4.8. Whole Genome Sequencing

Total DNA was isolated from *E. coli* BL21(DE3) cultures initiated each with a single colony. Whole genome sequencing was performed by Eurofins Genomics GmbH (Ebersberg, Germany) using Illumina sequencing technology. The quality of the obtained *fastq* files was checked using the software FastQC (Version 0.11.8) [36]. The reads were mapped to the reference genome of *E. coli* BL21 (DE3) (NC_012892.2) using the Burrows–Wheeler aligner with the “-mem” algorithm [37]. Subsequently, BCFtools (bcftools call -c) was used to call variants in the aligned reads [38]. The generated Binary Alignment Map and Variant Call Format files were further analyzed using the Integrative Genomics Viewer IGV 2.3 [12].

### 4.9. Construction of the Plasmid Expressing the Riboflavin Transporter Gene from C. glutamicum in E. coli

The synthetic *pnu*X gene coding for the riboflavin transporter from *C. glutamicum* cloned in pUC57 (pUC57-RT) was purchased from GeneScript (Piscataway, NJ, USA). For the expression of this gene in *E. coli,* the plasmid pREP4 was modified using the site directed mutagenesis by inverse PCR [34]. First, a DNA fragment carrying lacUV5 promoter and lac operator was inserted into pREP4 using Phusion high-fidelity DNA polymerase and a pair of primers, Rep-P and Rep-L, as depicted in Table S7. The resulting DNA ligation sample was transformed into *E. coli* XL1 cells. Clones containing the resulting plasmid pREPuv5 were identified with colony PCR using primers LacO and CP-R.

The *pnu*X was inserted into the pREPuv5 as depicted in Table S8, using Phusion high-fidelity DNA polymerase and pUC57-RT as a template. The resulting DNA ligation sample was transformed into *E. coli* XL1 cells. Clones that carried the resulting pREP-RT were identified with colony PCR using primers CgF2 and CgR1. The modified region of the pREP-RT was sequenced using primer CgS and the nucleotide, as well as the inferred amino acid sequences that are shown in the Figure S8.

### 4.10. Expression of the Avena Sativa LOV2 Protein, Isolation of Flavin Ligands, and Their Analysis with LC-MS

The LOV2 protein loaded with flavin was isolated and purified from a recombinant *E. coli* strain as described earlier [5]. The protein sample was transferred to the buffer 20 mmol/L Tris-HCl pH 8.0 by dialysis. Flavin ligands were isolated from the LOV2 by the addition of an equal volume of 100% methanol to the protein sample (10 mg/mL). The sample was incubated on ice for 30 min, centrifuged (16,200× *g*, 4 °C, 15 min), and the supernatant was saved.

Aliquots of the supernatant (10 µL) were analyzed using the Elute-ultra-high performance liquid chromatography (UHPLC) system (Bruker Daltonics, Bremen, Germany) equipped with RP C18 column (Kinetex, 150 mm × 2.1 mm, 1.7 µm, Phenomenex, Aschaffenburg, Germany) and coupled to a maXis ETD high-resolution electrospray ionization quadrupole time-of-flight (ESI-qTOF) mass spectrometer (Bruker Daltonics). The UHPLC was operated at a flow rate of 0.2 mL/min at 20 °C and the autosampler temperature was maintained at 5 °C. The mobile phases were water (A) and methanol/water (4:1, *v*/*v*) (B), both with 0.1% formic acid. The gradient of mobile phases was as follows: 0.0–2.0 min constant at 25% B, 2.0–17.0 min linear increase to 100% B, 17.0–24.0 min constant at 100% B, 24.0–25.0 min linear decrease to 25% B, and 25.0–30.5 min constant at 25% B. The mass spectrometer was operated in positive ionization mode, in the *m*/*z* range of 50 to 1000, with a spectral rate of 1 Hz. Ion source parameters were as follows: end plate offset −500 V, capillary 4500 V, nebulizer pressure 4.0 bar, dry gas 9.0 L/min, and dry temperature 200 °C. MS/MS experiments were carried out with collision energies of 7 and 20 eV. Collision experiments were conducted within the *m*/*z* range of 348 to 790. The calibration was performed using a solution of sodium formate (formic acid/1 M NaOH in water/isopropanol (0.1:1:100, *v*/*v*/*v*)). The calibrant mixture was injected once before the measurement series was started, as well as at the end of each run.

### 4.11. Cloning of the Dihydrolipoyl Dehydrogenase Gene lpdA from E. coli

*Lpd*A was amplified using primers lpdN1, lpdN2, lpdN3, and lpdC. A single colony of *E. coli* BL21(DE3)-Δ*rib*A freshly plated on LB agar was used as a source of DNA template. The exact procedure is depicted in Table S9. The resulting DNA fragment was treated with the restriction enzymes NdeI and XhoI, purified, and ligated with pET22b that was treated with the same restriction enzymes. DNA from the ligation sample was purified using the abovementioned kit and transformed into *E. coli* XL1 cells. Colonies carrying the plasmid with *lpd*A insert were identified by colony PCR using primers lpdN3 and lpdC. The *lpd*A region of the plasmid isolated from one positive clone was sequenced using standard sequencing primers T7 and pET-RP (Eurofins Genomics, Ebersberg, Germany).

## Figures and Tables

**Table 1:** Genomic regions (open reading frames or intergenic DNA fragments) where mutations were found in more than one evolution line. The empty cells in the columns “evolution line” 1, 2, or 3 indicate nucleotide sequences identical to those in the initial strain Δ*rib*A-RT.

Genome Coordinate According to NC_012892	Gene	Codon (Respective Amino Acid) in Reference Genome	Evolution Line			Protein
			1	2	3	
			Mutation (Amino Acid)			
131,146	*lpd*A	GCA (A144)	ACA (T)			dihydrolipoyl dehydrogenase
131,222		CTG (L169)			CAG (Q)	
131,795		GTG (V360)		GGG (G)		
991,109	*omp*F	GGT (G320)		TGT (C)		porin OmpF
991,602–991,607		CGT (R154) GTT (V155)	deleted			
991,877		CGT (R64)	TGT (C)			
991,942–991,953		GCT (A38) GTC (V39) GGT (G40) CTG (L41)			deleted	
4,176,743	IR ^1^	C	T	T		NA ^2^

^1^ The mutation is located in the intergenic region 74 bp upstream from the *aph*A (coding for acid phosphatase) and 328 bp upstream from the ORF B21_RS2502 (coding for protein YjbS). ^2^ Not applicable.

**Table 2:** Relative growth values as the quotient of the OD_600_ value for the culture with **8** to the OD_600_ value of the culture without **8**, of the Δ*rib*A-RT strain carrying plasmid with wild-type *lpd*A (WT), or with one of the mutant genes. Cultivations of each strain were carried out in triplicate.

Strain	*lpd*A Variant	Cultivation			Quotient Mean Value ± sd
		1	2	3	
1	WT	0.98	1.01	0.82	0.94 ± 0.08
2	A144T	1.12	1.15	1.07	1.11 ± 0.03 ^a^
3	L169Q	1.34	1.07	1.08	1.16 ± 0.13
4	V360G	1.19	1.20	1.55	1.31 ± 0.17 ^a^

^a^ According to the Student’s *t*-test, the sample population differs significantly (*p* < 0.05) from the WT sample population.

## Data Availability

Data presented in this study are available within the article.

## Supplementary Materials

The following supporting information can be downloaded at: https://www.mdpi.com/article/10.3390/molecules29245891/s1, Figure S1: Nucleotide sequence of the *rib*A and protein sequence of the GTP-cyclohydrolase II from *E. coli*; Figure S2: Nucleotide sequence of the *rib*A region after CRISPR-Cas9-assisted genome editing of *E. coli* BL21(DE3); Figure S3: LC-MS chromatogram of flavins isolated from LOV2 protein that was produced in *E. coli*-Δ*rib*A cells fed with the mix of riboflavin (1.9 µmol/L) and 7,8-dideoxyriboflavin (3.0 µmol/L); Figure S4: Absorbency at 600 nm (blue bars) and riboflavin concentration in cultivation medium (red line) of the evolution lines 2 (A) and 3 (B); Figure S5: Aligned nucleotide sequences of the *lpd*A clones: WT (initial *E. coli* strain); EL1 – EL3, evolution lines 1 to 3 (passage number 100). SNPs are shown in cyan; Figure S6: Aligned protein sequences inferred from the nucleotide sequences of the *lpd*A clones: WT (initial strain), El1 to EL3: evolution lines 1 to 3 (passage number 100). Figure S7: RP18-HPLC chromatograms of the samples of riboflavin, 6, 7 and 8 used for the evolution experiment; Figure S8: Nucleotide sequence of the region of pREP-RT containing lacUV5 promoter (in yellow), lac operator (in cyan) and riboflavin transporter gene; Table S1: The editing template ETribA for the knockout of *E. coli* BL21(DE3)-Δ*rib*A; Table S2: Primers used in this study; Table S3: Mutations that were identified in three clones isolated from three evolution lines that are absent in the starting *E. coli* strain (∆*rib*A-RT); Table S4: Bacterial strains and plasmids used in this study; Table S5: Construction of pTargetF-*rib*A carrying gene for *rib*A-sgR; Table S6: Synthesis of the editing template ETribA. Table S7: Preparation of pREP-uv5 on the basis of pREP-4 vector; Table S8: Preparation of pREP-RT on the basis of pREP-uv5 vector; Table S9: Amplification of the *lpd*A DNA fragment from *E. coli*. References [39,40] are cited in the Supplementary Materials.
