# Supplementary material for: Adaptive Laboratory Evolution of Flavin Functionality Identifies Dihydrolipoyl Dehydrogenase as One of the Critical Points for the Activity of 7,8-Didemethyl-Riboflavin as a Surrogate for Riboflavin in Escherichia coli
Source: Molecules. 2024 Dec 13;29(24):5891. doi: 10.3390/molecules29245891 (PMC11677807; doi:10.3390/molecules29245891)
Supplement: Supplementary file 1 [file molecules-29-05891-s001.zip › molecules-3306105-supplementary.pdf]

Supporting Materials

Adaptive laboratory evolution of flavin functionality identified dihydrolipoyl dehydrogenase as one of the critical points for activity of 7,8-didemethyl-riboflavin as a surrogate for riboflavin in *Escherichia coli*.

Farshad La-Rostami[1], Alexandra Scharf[1], Chenyang Albert[1], Nils Wax[1], Marina Creydt,[1] Boris Illarionov[1], Adelbert Bacher[2], Stefan Weber[3], Markus Fischer[1,*]

[1] Hamburg School of Food Science, Institute of Food Chemistry, University of Hamburg, Grindelallee 117, 20146 Hamburg, Germany

[2] TUM School of Natural Sciences, Technical University of Munich, Boltzmannstraße 10, 85748 Garching, Germany

[3] Institute of Physical Chemistry, Albert-Ludwigs-Universität Freiburg, Albertstraße. 21, 79104 Freiburg, Germany

1. *Preparation of E. coli BL21(DE3)-ΔribA strain*

Protospacer regions with high GC content or poly-T sequences were avoided. The selected target sequence for the knockout of the *rib*A in *E. coli* BL21(DE3) was 5'-GCCAAACTGCCAACCCCAT<u>GGG</u>-3' (protospacer adjacent motif or PAM sequence is underlined) (positions 25 to 46 in the *rib*A open reading frame, Figure S1). For the inactivation of *rib*A*,* the DNA fragment of 23 bp (nucleotides 31 to 53) was chosen for replacement with a synthetic DNA fragment, as shown in Table S1. For this purpose, a pTargetF-*rib*A plasmid with the *rib*A-sgRNA coding DNA fragment as well as the *rib*A editing template were constructed as described in the Materials and Methods section. The resulting editing template was 811 bp long and had two DNA arms of 400 bp and 396 bp, identical to the DNA regions located upstream or downstream from the protospacer region in the *rib*A with a 15 bp artificial DNA fragment in between (Table S1).

The plasmid pTarget-*rib*A and the DNA fragment ETribA were transformed together into *E. coli* BL21(DE3) harboring pCas. The riboflavin auxotrophs were identified by replating the resulting colonies from LB agar containing riboflavin (200 mg/L) onto LB agar without riboflavin. Several clones auxotrophic for riboflavin were tested for the presence of the 15 bp artificial DNA fragment in the ribA by colony polymerase chain reaction (PCR). Primers Amr1 (which binds at the C-terminal end of the ribA) and ribA-mutfw (binds the artificial DNA fragment inserted into ribA) (see Table S2) were used for this purpose. A small part of a single E. coli colony taken from a fresh LB plate was used as the DNA source. Denaturation of DNA was carried out at 98 °C for 5 seconds, annealing at 60 °C for 15 seconds, and polymerization at 72 °C for 45 seconds. If no artificial insertion DNA fragment is present, no amplification takes place. While no DNA bands for any of the tested negative controls (BL21(DE3) strain) were visible in the agarose gel after electrophoresis, all tested riboflavin-auxotrophic clones showed DNA fragments of the expected size. A DNA fragment from one positive clone was generated by PCR using primers AmL1 + AmR1. The nucleotide sequence of the amplified DNA fragment was determined using Sanger sequencing and confirmed the insertion of the 15 bp artificial DNA fragment, which resulted in the removal of the PAM sequence, a frameshift, and 16 stop codons in the *rib*A gene (Figure S2).

2. *Determination of flavin concentration*

The concentration of riboflavin or demethyl-riboflavin species **6**, **7,** or **8** was determined photometrically using a spectrophotometer Ultrospec 4300 pro (Lab Logistics Group GmbH, Meckenheim, Germany). The molar absorption coefficient (ε_{470}) for riboflavin is 9600 M^{-1} cm^{-1}, for **6** is 6100 $M^{-1} \cdot cm^{-1}$, for **7** is 9600 $M^{-1} \cdot cm^{-1}$, and for **8** is 6500 $M^{-1} \cdot cm^{-1}$.

3. *Conducting adaptive evolution experiment*

Approximately every 10 passages, 0.5 mL from each evolution line was centrifuged, resuspended in 0.5 mL of M9 medium with 30% glycerol, and stored at -80 °C (stock culture). The latter were used for the recovery of a particular evolution line when it must have been interrupted due to, e.g., contamination with other microorganisms. Additionally, for culture purity testing, every 15 to 20 passages, several microliters from each culture were streaked on LB agar containing Km (20 mg/L). Colony PCR was performed on five grown colonies using the primers AmL1 and AmC (Table S2) to confirm that these were *E. coli* cells that contained the deletion in *rib*A. Furthermore, ten to twelve single colonies from the same plate were replated on M9 agar without flavins in order to check if they became prototrophic for riboflavin. In parallel to the three evolution lines grown with flavins, one blank culture in growth medium without any flavin was run. The latter was inoculated with 1.5 µL from each of the three evolution lines of the previous passage and served as an indicator for the appearance in the evolution lines of microorganisms prototrophic for riboflavin. When, after 24 hours of incubation the OD_{600} in the blank culture exceeded the value of 0.3, the evolution line with an OD_{600} value above 1.5 in this passage was discarded and replenished from the last stock culture.

Tables

Table S1. The editing template ETribA for the knockout of *E. coli* BL21(DE3)-Δ*rib*A. Nucleotide sequences corresponding to the flanking primers AmL1 and AmR1 are underlined; those corresponding to the internal primers AmL2 and AmR2 are in shaded grey. The artificial DNA fragment that replaced the target protospacer region in *rib*A is shown in lowercase letters.

Nucleotide sequence (5'→ 3')	
ACACCAGAGAAACCAGCCGAATAAAATCAAATGTGTAATGACGCCCCAGG	50
GCTGGGTGACAGTTTCAGTAACCCAAAAAGCCGCTTTTAGTAGCCAACTT	100
TGTTCTCCAGGTTGCCAACGCCAGCCAGAAATCCATACGGCTACTGGCAT	150
GACAAGCAATAGTGCAGCTCCCACTGCGGTACGTCTGGCAATCGAACGCA	200
TGGCCTCTCCTTTTTGATAAGTCCCACAATCATAACTGAAAACGCCAGTT	250
CCAGGAAAAATTGACAGATTTGTGCCATTCCGTGAACGATCGACGCGTCG	300
TGATTAGGTGAACCCCTTCTCGTTATGGCAAAATAAGCCAATACAGAACC	350
AGCATTATCTGGAGAATTTCATGCAGCTTAAACGTGTGGCAGAAGCCTAA	400
ggaccgcacgtgtgaCCTGATGGTGGGATTTGAAGAACTGGCAACCGGAC	450
ACGATCATGTCGCGCTAGTCTATGGCGATATTTCCGGGCATACCCCGGTA	500
CTTGCGCGCGTCCATTCCGAATGTCTGACCGGTGACGCCCTGTTCAGCTT	550
GCGCTGCGATTGTGGCTTCCAGCTCGAAGCGGCATTGACGCAAATTGCCG	600
AGGAAGGCCGTGGTATTTTGCTGTATCACCGTCAGGAAGGTCGTAACATT	650
GGTCTGCTGAATAAAATCCGCGCTTACGCACTGCAGGATCAAGGTTACGA	700
TACCGTAGAGGCTAACCACCAGTTAGGCTTCGCCGCTGATGAGCGCGACT	750
TCACTCTTTGCGCTGATATGTTCAAACTCCTTGGCGTCAATGAAGTCCGC	800
TTGTTAACCAA	811

Table S2. Primers used in this study.

Primer	Primer sequence (5' → 3') [a]	Application area
ribAfw1	AAACTGCCAACCCCATGGTTTTAGAGCTAGAAATAGCAAGTTAAAAT	Construction of pTargetF-*rib*A carrying the DNA fragment coding for *rib*A-sgRNA and colony PCR screening
ribAfw2	GAGAACTAGTCAGAAGCCAAACTGCCAACCCCATG	
ribAr	TCTCAGAATTCAAAAAAAGCACCGA	
AmL1	ACACCAGAGAAACCAGCC	Synthesis of the editing template ETribA for knockout of *rib*A in *E. coli*. Colony PCR screening.
AmL2	TCACACGTGCGGTCCTTAGGCTTCTGCCACACG	
AmR1	TTGGTTAACAAGCGGACTTC	
AmR2	GGACCGCACGTGTGACCTGATGGTGGGATTTGA	
AmC	TCACACGTGCGGTCC	
ribA-mutfw	GGACCGCACGTGTGA	Colony PCR screening
pTargetFr	GTAAGCACTACATTTCGCTC	Sanger sequencing
Rep-P	TGCCTCGAGCTCGTATAATATGTGGAATTGTGAGCGGATAACAATTTAATCTAGATTTCAGTGCAATTTATCTCTTCAAA	Construction of pREP-uv5
Rep-L	GAGCTCGAGGCATAAAGTGTAAAATAATATTTTATCTGATTAATAAGATGATCTTCTTGA	
LacO	TGTGGAATTGTGAGCGG	
CP-R	ACCGTTTCTGCGGAC	
CgF1	AATTAACCATGAATCCTATAACCGAATTATTAGACGCAACATTATG	Construction of pREP-RT
CgF2	ACATCTAGAAAAGAGGAGAAATTAACCATGAATCCTATAACCGAATTATTAGACGCAACATTATG	
CgR1	GGAAAGCTTCAGACTGTCACAGACTCTTGAGCTTCC	
CgS	TAGCTGACATTCATC	
lpdN1	GGAAAACCTGTACTTCAGCAGTACTGAAATCAAAACTCAGG	Cloning of the *lpd*A gene of *E. coli*.
lpdN2	CACCATCACCATGGTCTGGAAAACCTGTACTTCAGCAGT	
lpdN3	GAGATCCATATGCACCATCACCATCACCATGGTGCGGA	
lpdC	GAGATCCTCGAGTTACTTCTTCTTCGCTTTCGGG	

[a] Restriction sites used for cloning or plasmid construction are underlined.

Table S3. Mutations that were identified in three clones isolated from three evolution lines that are absent in the starting *E. coli* strain (∆*rib*A-RT). Nucleotide positions according to the NCBI record NC_012892.

**Evolution line**		**1**	**2**	**3**
**Position**	**Nucleotide or nucleotide sequence in the starting ∆*rib*A-RT strain**	**Nucleotide substitution in the respective evolution line [a]**		
34054	CCGATATCGATA			CCGATA
124429	T			C
131146	G	A		
131222	T			A
131795	T		G	
200753	C			G
201925	GCGTCTGAATCGTCTG			GCGTCTG
290335	C		T	
439169	TCC	TC		
991109	C		A	
991601	CAACACGA	CA		
991877	G	A		
991942-991953	GCAGACCGACAGCT			GT
1009815	GCC		GC	
1629794	G	A		
2138903	TC			TCC
2352940	G			C
2352954	T			C
2353027	T			C
2651240	GTT			GTTT
3211736	T			A
3933035	C	T		
4176743	C	T	T	
**Total number of mutations per evolution line**		7	5	12

[a] The empty chart cell means there is no difference with the nucleotide sequence of the starting ∆*rib*A-RT strain

Table S4. Bacterial strains and plasmids used in this study.

Bacterial strain or plasmid	Relevant characteristics and application area	Reference and / or source
E. coli **XL1**	Cloning of DNA fragments and construction of plasmids	Bullock, 1987 [1]
E. coli **BL21(DE3)**	Carries T7 RNA polymerase and *lacI* genes. Used for CRISPR-Cas9 based genome editing.	Studier and Moffatt, 1986 [2]
E. coli **BL21(DE3) Δ*rib*A**	Riboflavin auxotroph carrying deletion in *rib*A.	This study
E. coli **BL21(DE3) Δ*rib*A[pREP-RT]; further referred as Δ*rib*A-RT**	Riboflavin auxotroph that expresses recombinant riboflavin transporter gene of *C. glutamicum*. Bacterial strain used in laboratory evolution experiments.	This study
**pCas**	Part of the CRISPR-Cas9 based two-plasmid system for genome editing of *E. coli.* Carries *cas*9 gene, λ-Red DNA recombination genes, sgRNA gene targeting the pTargetF pMB1 replicon. Kanamycin resistance.	Jiang et al., 2015 [8] / Addgene (accession 62225)
**pTargetF**	Part of the CRISPR-Cas9 based two-plasmid system for genome editing of *E. coli.* Spectinomycin resistance.	Jiang et al., 2015 [8] / Addgene (accession 62226)
**pTargetF-*rib*A**	Part of the CRISPR-Cas9 based two-plasmid system for genome editing of *E. coli*. Expression of *rib*A-sgRNA. Spectinomycin resistance.	This study
**pREP-uv5**	pREP4 based plasmid carrying lacUV5 promoter. Expression of genes controlled by lacUV5.	This study
**pREP-RT**	pREP4 based plasmid carrying lacUV5 promoter and the riboflavin transport gene from *Corynebacterium glutamicum.* Transport of riboflavin and riboflavin analogs from growth medium into *E. coli* cell. Kanamycin resistance.	This study
**pT7-HISLOVC450A**	pT7 plasmid carrying the DNA fragment coding for the LOV2 domain of *Avena sativa*. Verification of transport of deoxyriboflavin analogs from growth medium into *E. coli* cells by riboflavin transporter. Ampicillin resistance.	Richert et al., 2019 [5]
**pET-lpdAm0**	pET22b(+) carrying *E. coli* wild-type *lpd*A gene.	This study
**pET-lpdAm1**	pET22b(+) carrying mutated *E. coli lpd*A gene (A144$\rightarrow$T) isolated from evolution line 1.	This study
**pET-lpdAm2**	pET22b(+) carrying mutated *E. coli lpd*A gene (L169$\rightarrow$Q) isolated from evolution line 3) .	This study
**pET-lpdAm3**	pET22b(+) carrying mutated *E. coli lpd*A gene (V360$\rightarrow$G) isolated from evolution line 2.	This study

Table S5. Construction of pTargetF-*ribA* carrying gene for *rib*A-sgRNA. Each amplification step ended with incubation at 72 °C for 10 minutes. Primer specifications are shown in Table S2.

Amplifi-cation step	Primers	Template	Denaturation, T °C / sec	Annealing, T °C / sec	Polymerization, T °C / sec	Number of cycles
1	ribAfw1 + ribAr	pTargetF, 20 ng	98 / 5	60 / 15	72 / 45	10
PCR product was transferred to 50 µl of water using Monarch PCR and DNA cleanup kit						
2	ribAfw2 + ribAr	2 µl of the PCR product from the step 1	98 / 5	60 / 15	72 / 45	25
PCR product was transferred to 200 µl of water using Monarch PCR and DNA cleanup kit, treated with restriction endonuclease SpeI, transferred to 200 µl of water, treated with T4 DNA ligase, transferred to 50 µl of water, and stored at -20 °C.						

Table S6. Synthesis of the editing template ETribA. Each amplification step ended with incubation at 72 °C for 10 minutes. Primer specifications are shown in Table S2.

Amplifica-tion step	Primers	Template	Denaturation, T °C / sec	Annealing, T °C / sec	Polymerization, T °C / sec	Number of cycles
1	AmL1 + AmL2	Genomic DNA *E. coli*, ~20 ng	98 / 5	60 / 15	72 / 10	15
PCR product was transferred to 50 µl of water using Monarch PCR and DNA cleanup kit						
2	AmR1 + AmR2	Genomic DNA *E. coli*, ~20 ng	98 / 5	60 / 15	72 / 10	15
PCR product was transferred to 50 µl of water using Monarch PCR and DNA cleanup kit						
3	AmL1 + AmR1	2 µl PCR product step 1 + 2 µl PCR product step2	98 / 5	60 / 15	72 / 20	25
PCR product was purified from agarose gel using peqGOLD gel extraction kit, dissolved in 50 µl of water, and stored at -20 °C.						

Table S7. Preparation of pREP-uv5 on the basis of pREP-4 vector. Each amplification step ended with incubation at 72 °C for 10 minutes. Primer specifications are shown in Table S2.

Amplifica-tion step	Primers	Template	Denaturation, T °C / sec	Annealing, T °C / sec	Polymerization, T °C / sec	Number of cycles
1	REP-L + REP-P	pREP-4, 20 ng	98 / 5	60 / 15	72 / 70	25
PCR product was purified using peqGOLD gel extraction kit, treated with XhoI restriction enzyme, purified as mentioned above, treated with T4 DNA ligase, purified, and stored at -20 °C.						

Table S8. Preparation of pREP-RT on the basis of pREP-uv5 vector. Each amplification step ended with incubation at 72 °C for 10 minutes. Primer specifications are shown in Table S2.

Amplifica-tion step	Primers	Template	Denaturation, T °C / sec	Annealing, T °C / sec	Polymerization, T °C / sec	Number of cycles
1	CgF1 + CgR1	pREP-uv5, 20 ng	98 / 5	66 / 15	72 / 35	10
PCR product was transferred to 50 µl of water using Monarch PCR and DNA cleanup kit.						
2	CgF2 + CgR1	2 µl PCR product step 1	98 / 5	68 / 15	72 / 35	25
PCR product was purified from agarose gel using peqGOLD gel extraction kit, dissolved in 50 µl of water, treated with restriction enzymes XbaI and HindIII, purified, and ligated with pREP-uv5 treated with the same enzymes. The ligation mix was transferred into 50 µl of water with peqGold cyclo pure kit and stored at -20 °C.						

Table S9. Amplification of the *lpd*A DNA fragment from *E. coli.* Each amplification step ended with incubation at 72 °C for 10 minutes.

Amplificati on step	Primers	Template	Denaturation, T °C / sec	Annealing, T °C / sec	Polymerization, T °C / sec	Number of cycles
1	lpdN1 + lpdC	Genomic DNA from *E. coli* BL21(DE3)	98 / 5	62 / 15	72 / 120	5
PCR product was purified and dissolved in 50 µl of water						
2	lpdN2 + lpdC	2 µl of PCR product from the step 1	98 / 5	62 / 15	72 / 120	5
PCR product was purified and dissolved in 50 µl of water						

3	lpdN3 + lpdC	2 µl of PCR product from the step 2	98 / 5	62 / 15	72 / 120	25

PCR product was purified from agarose gel using peqGOLD gel extraction kit, dissolved in 50 µl of water, and stored at -20 °C.

Figures

```
  1   ATGCAGCTTAAACGTGTGGCAGAAGCCAAACTGCCAACCCCATGGGGCGATTTCCTGATG
       M  Q  L  K  R  V  A  E  A  K  L  P  T  P  W  G  D  F  L  M

 60   GTGGGATTTGAAGAACTGGCAACCGGACACGATCATGTCGCGCTAGTCTATGGCGATATT
       V  G  F  E  E  L  A  T  G  H  D  H  V  A  L  V  Y  G  D  I

120   TCCGGGCATACCCCGGTACTTGCGCGCGTCCATTCCGAATGTCTGACCGGTGACGCCCTG
       S  G  H  T  P  V  L  A  R  V  H  S  E  C  L  T  G  D  A  L

180   TTCAGCTTGCGCTGCGATTGTGGCTTCCAGCTCGAAGCGGCATTGACGCAAATTGCCGAG
       F  S  L  R  C  D  C  G  F  Q  L  E  A  A  L  T  Q  I  A  E

240   GAAGGCCGTGGTATTTTGCTGTATCACCGTCAGGAAGGTCGTAACATTGGTCTGCTGAAT
       E  G  R  G  I  L  L  Y  H  R  Q  E  G  R  N  I  G  L  L  N

300   AAAATCCGCGCTTACGCACTGCAGGATCAAGGTTACGATACCGTAGAGGCTAACCACCAG
       K  I  R  A  Y  A  L  Q  D  Q  G  Y  D  T  V  E  A  N  H  Q

360   TTAGGCTTCGCCGCTGATGAGCGCGACTTCACTCTTTGCGCTGATATGTTCAAACTCCTT
       L  G  F  A  A  D  E  R  D  F  T  L  C  A  D  M  F  K  L  L

420   GGCGTCAATGAAGTCCGCTTGTTAACCAATAACCCGAAAAAAGTCGAAATTCTGACCGAA
       G  V  N  E  V  R  L  L  T  N  N  P  K  K  V  E  I  L  T  E

480   GCAGGGATTAATATTGTTGAACGCGTACCATTGATTGTAGGTCGTAACCCCAATAACGAA
       A  G  I  N  I  V  E  R  V  P  L  I  V  G  R  N  P  N  N  E

540   CATTATCTCGATACCAAAGCCGAGAAAATGGGCCATTTGCTGAACAAATAA
       H  Y  L  D  T  K  A  E  K  M  G  H  L  L  N  K  *
```

Figure S1. Nucleotide sequence of the *rib*A and protein sequence of the GTP-cyclohydrolase II from *E. coli*. The target sequence (protospacer region) selected for genome editing is underlined. The DNA fragment deleted in the *rib*A knockout is highlighted in gray. The PAM sequence is shown in box. Amino acid residues that are critical for the enzyme function are shadowed gray.

```
  1   ATGCAGCTTAAACGTGTGGCAGAAGCCTAAggaccgcacgtgtgaCCTGATGGTGGGATT
       M  Q  L  K  R  V  A  E  A  *  G  P  H  V  *  P  D  G  G  I
 60   TGAAGAACTGGCAACCGGACACGATCATGTCGCGCTAGTCTATGGCGATATTTCCGGGCA
       *  R  T  G  N  R  T  R  S  C  R  A  S  L  W  R  Y  F  R  A
120   TACCCCGGTACTTGCGCGCGTCCATTCCGAATGTCTGACCGGTGACGCCCTGTTCAGCTT
       Y  P  G  T  C  A  R  P  F  R  M  S  D  R  *  R  P  V  Q  L
180   GCGCTGCGATTGTGGCTTCCAGCTCGAAGCGGCATTGACGCAAATTGCCGAGGAAGGCCG
       A  L  R  L  W  L  P  A  R  S  G  I  D  A  N  C  R  G  R  P
240   TGGTATTTTGCTGTATCACCGTCAGGAAGGTCGTAACATTGGTCTGCTGAATAAAATCCG
       W  Y  F  A  V  S  P  S  G  R  S  *  H  W  S  A  E  *  N  P
300   CGCTTACGCACTGCAGGATCAAGGTTACGATACCGTAGAGGCTAACCACCAGTTAGGCTT
       R  L  R  T  A  G  S  R  L  R  Y  R  R  G  *  P  P  V  R  L
360   CGCCGCTGATGAGCGCGACTTCACTCTTTGCGCTGATATGTTCAAACTCCTTGGCGTCAA
       R  R  *  *  A  R  L  H  S  L  R  *  Y  V  Q  T  P  W  R  Q
420   TGAAGTCCGCTTGTTAACCAATAACCCGAAAAAAGTCGAAATTCTGACCGAAGCAGGGAT
       *  S  P  L  V  N  Q  *  P  E  K  S  R  N  S  D  R  S  R  D
480   TAATATTGTTGAACGCGTACCATTGATTGTAGGTCGTAACCCCAATAACGAACATTATCT
       *  Y  C  *  T  R  T  I  D  C  R  S  *  P  Q  *  R  T  L  S
540   CGATACCAAAGCCGAGAAAATGGGCCATTTGCTGAACAAATAA
       R  Y  Q  S  R  E  N  G  P F  A  E  Q  I
```

Figure S2. Nucleotide sequence of the *rib*A region after CRISPR-Cas9-assisted genome editing of *E. coli* BL21(DE3). The artificial DNA fragment inserted into the *rib*A gene is shown in lowercase letters. Stop codons are indicated by asterisks. The single mutation (A $\rightarrow$ T) that generated the first stop codon in the *rib*A open reading frame is shadowed.

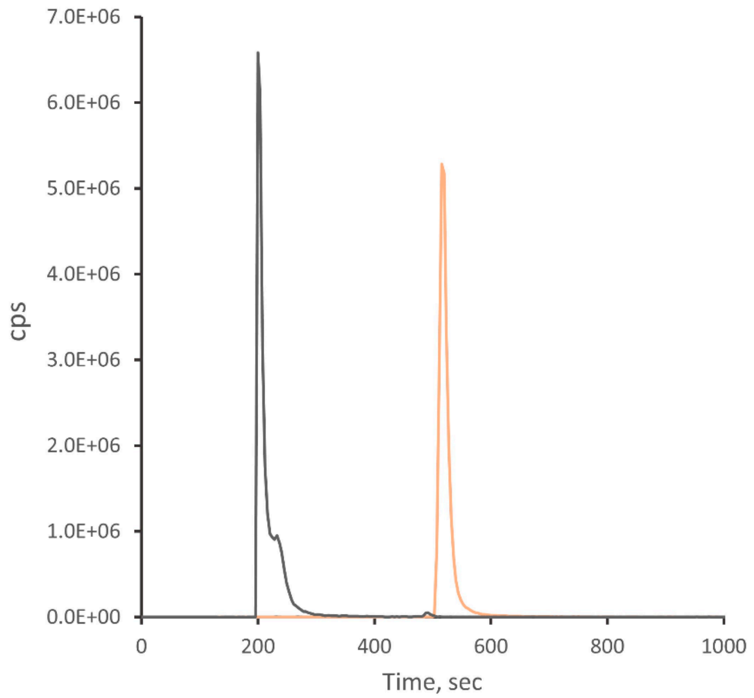

Figure S3. LC-MS chromatogram of flavins isolated from the LOV2 protein that was produced in *E. coli*-Δ*rib*A cells fed with the mix of riboflavin (1.9 µmol/L) and 7,8-dideoxyriboflavin (3.0 µmol/L). FMN (m/z 457.1119), yellow track; 7,8-dideoxyFMN (m/z 429.0806), gray track.

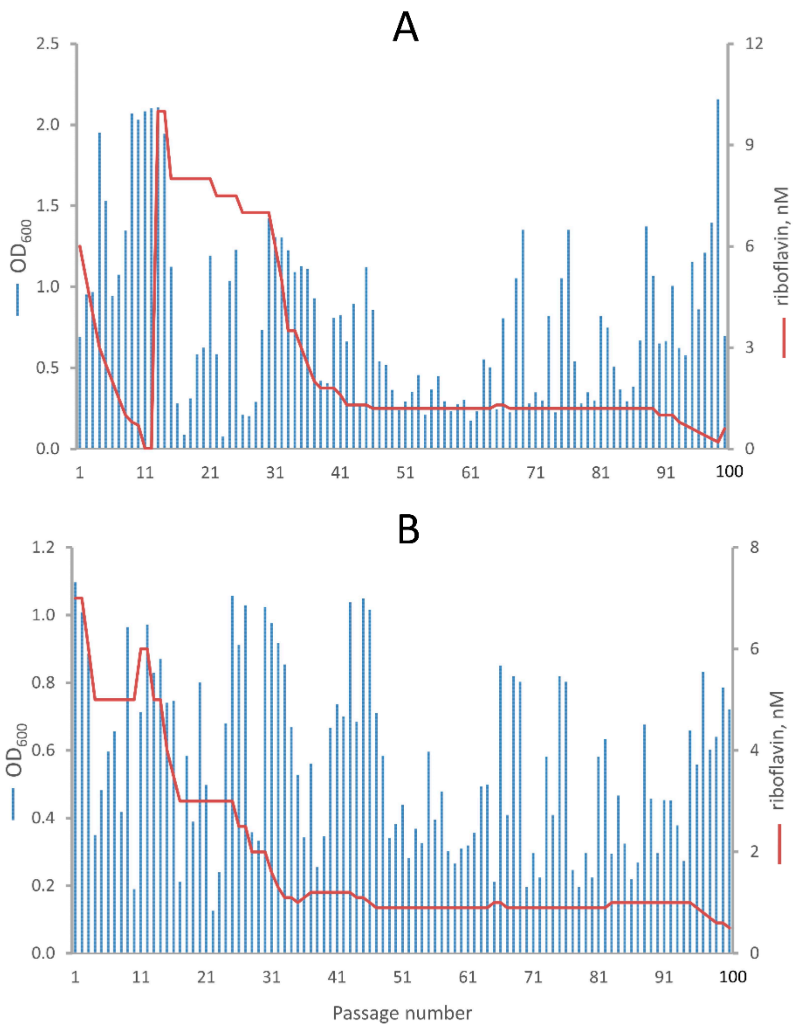

Figure S4. Absorbency at 600 nm (blue bars) and riboflavin concentration in cultivation medium (red line) of the evolution lines 2 (**A**) and 3 (**B**).

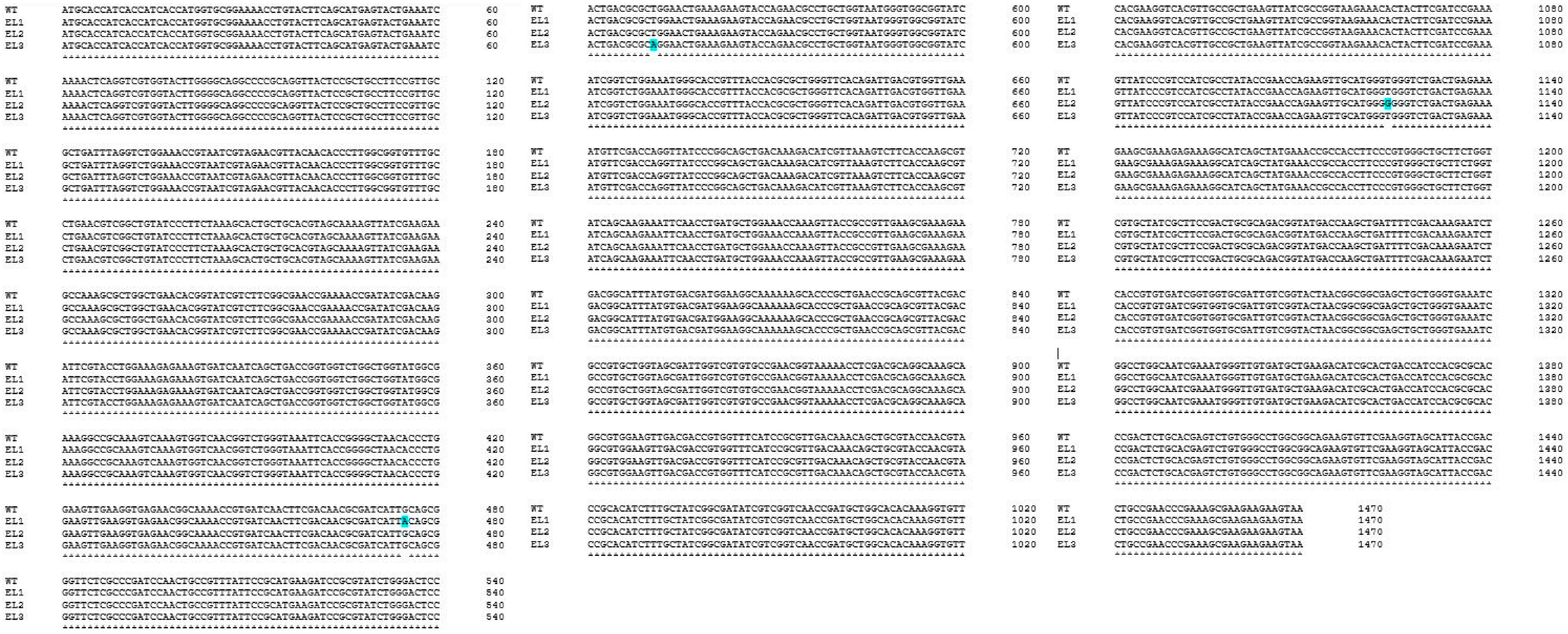

Figure S5. Aligned nucleotide sequences of the *lpd*A clones: WT (initial *E. coli* strain); EL1 – EL3, evolution lines 1 to 3 (passage number 100). SNPs are shown in cyan.

```
WT      MHHHHHHGAENLYFSMSTEIKTQVVVLGAGPAGYSAAFRCADLGLETVIVERYNTLGGVC    60
EL1     MHHHHHHGAENLYFSMSTEIKTQVVVLGAGPAGYSAAFRCADLGLETVIVERYNTLGGVC    60
EL2     MHHHHHHGAENLYFSMSTEIKTQVVVLGAGPAGYSAAFRCADLGLETVIVERYNTLGGVC    60
EL3     MHHHHHHGAENLYFSMSTEIKTQVVVLGAGPAGYSAAFRCADLGLETVIVERYNTLGGVC    60
        ************************************************************

WT      LNVGCIPSKALLHVAKVIEEAKALAEHGIVFGEPKTDIDKIRTWKEKVINQLTGGLAGMA    120
EL1     LNVGCIPSKALLHVAKVIEEAKALAEHGIVFGEPKTDIDKIRTWKEKVINQLTGGLAGMA    120
EL2     LNVGCIPSKALLHVAKVIEEAKALAEHGIVFGEPKTDIDKIRTWKEKVINQLTGGLAGMA    120
EL3     LNVGCIPSKALLHVAKVIEEAKALAEHGIVFGEPKTDIDKIRTWKEKVINQLTGGLAGMA    120
        ************************************************************

WT      KGRKVKVVNGLGKFTGANTLEVEGENGKTVINFDNAIIAAGSRPIQLPFIPHEDPRIWDS    180
EL1     KGRKVKVVNGLGKFTGANTLEVEGENGKTVINFDNAIITAGSRPIQLPFIPHEDPRIWDS    180
EL2     KGRKVKVVNGLGKFTGANTLEVEGENGKTVINFDNAIIAAGSRPIQLPFIPHEDPRIWDS    180
EL3     KGRKVKVVNGLGKFTGANTLEVEGENGKTVINFDNAIIAAGSRPIQLPFIPHEDPRIWDS    180
        ************************************** *********************

WT      TDALELKEVPERLLVMGGGIIGLEMGTVYHALGSQIDVVEMFDQVIPAADKDIVKVFTKR    240
EL1     TDALELKEVPERLLVMGGGIIGLEMGTVYHALGSQIDVVEMFDQVIPAADKDIVKVFTKR    240
EL2     TDALELKEVPERLLVMGGGIIGLEMGTVYHALGSQIDVVEMFDQVIPAADKDIVKVFTKR    240
EL3     TDAQELKEVPERLLVMGGGIIGLEMGTVYHALGSQIDVVEMFDQVIPAADKDIVKVFTKR    240
        *** ********************************************************

WT      ISKKFNLMLETKVTAVEAKEDGIYVTMEGKKAPAEPQRYDAVLVAIGRVPNGKNLDAGKA    300
EL1     ISKKFNLMLETKVTAVEAKEDGIYVTMEGKKAPAEPQRYDAVLVAIGRVPNGKNLDAGKA    300
EL2     ISKKFNLMLETKVTAVEAKEDGIYVTMEGKKAPAEPQRYDAVLVAIGRVPNGKNLDAGKA    300
EL3     ISKKFNLMLETKVTAVEAKEDGIYVTMEGKKAPAEPQRYDAVLVAIGRVPNGKNLDAGKA    300
        ************************************************************

WT      GVEVDDRGFIRVDKQLRTNVPHIFAIGDIVGQPMLAHKGVHEGHVAAEVIAGKKHYFDPK    360
EL1     GVEVDDRGFIRVDKQLRTNVPHIFAIGDIVGQPMLAHKGVHEGHVAAEVIAGKKHYFDPK    360
EL2     GVEVDDRGFIRVDKQLRTNVPHIFAIGDIVGQPMLAHKGVHEGHVAAEVIAGKKHYFDPK    360
EL3     GVEVDDRGFIRVDKQLRTNVPHIFAIGDIVGQPMLAHKGVHEGHVAAEVIAGKKHYFDPK    360
        ************************************************************

WT      VIPSIAYTEPEVAWVGLTEKEAKEKGISYETATFPWAASGRAIASDCADGMTKLIFDKES    420
EL1     VIPSIAYTEPEVAWVGLTEKEAKEKGISYETATFPWAASGRAIASDCADGMTKLIFDKES    420
EL2     VIPSIAYTEPEVAWGGLTEKEAKEKGISYETATFPWAASGRAIASDCADGMTKLIFDKES    420
EL3     VIPSIAYTEPEVAWVGLTEKEAKEKGISYETATFPWAASGRAIASDCADGMTKLIFDKES    420
        ************** *********************************************

WT      HRVIGGAIVGTNGGELLGEIGLAIEMGCDAEDIALTIHAHPTLHESVGLAAEVFEGSITD    480
EL1     HRVIGGAIVGTNGGELLGEIGLAIEMGCDAEDIALTIHAHPTLHESVGLAAEVFEGSITD    480
EL2     HRVIGGAIVGTNGGELLGEIGLAIEMGCDAEDIALTIHAHPTLHESVGLAAEVFEGSITD    480
EL3     HRVIGGAIVGTNGGELLGEIGLAIEMGCDAEDIALTIHAHPTLHESVGLAAEVFEGSITD    480
        ************************************************************

WT      LPNPKAKKK        489
EL1     LPNPKAKKK        489
EL2     LPNPKAKKK        489
EL3     LPNPKAKKK        489
        *********
```

Figure S6. Aligned protein sequences inferred from the nucleotide sequences of the *lpd*A clones: WT (initial strain), El1 to EL3: evolution lines 1 to 3 (passage number 100). Single amino acid replacements are shown in cyan.

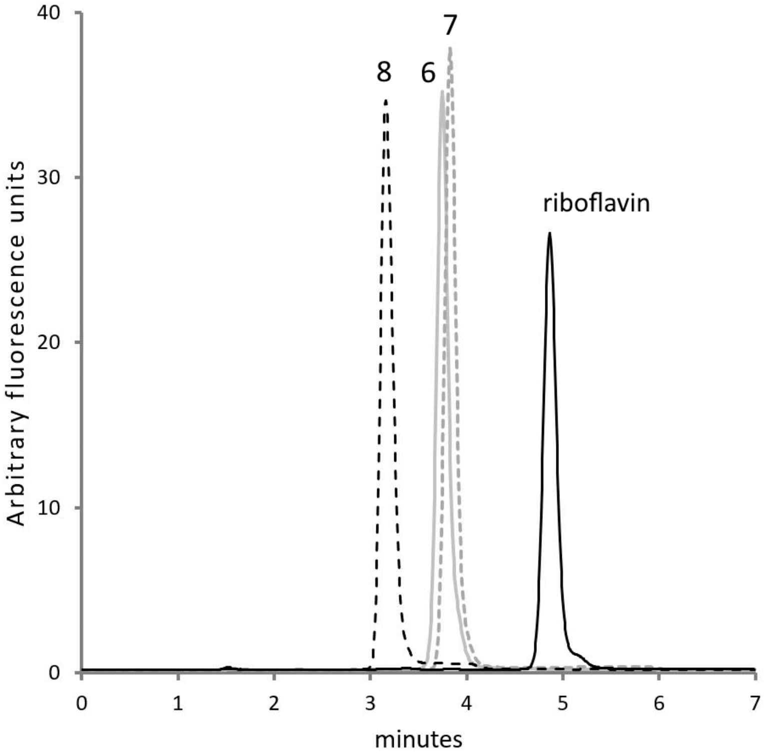

Figure S7. RP18-HPLC chromatograms of the samples of riboflavin, **6**, **7,** and **8** used for the evolution experiment.

```
AGGACAAGTTTTTGGTGACTGCGCTCCTTCCAAGCCAGTTACCTCGGTTCAAAAGAGTTG

GTAGCTCAGAGAACCTTCGAAAAACCGCCCTGCAAGGCGGTTTTTCGTTTTCAGAGCAA
                                           AseI
GAGATTACGCGCAGACCAAAACGATCTCAAGAAGATCATCTTATTAATCAGATAAAATAT
    -35           XhoI SacI   -10          lac-operator
TATTTTACACTTTATGCCTCGAGCTCGTATAATATGTGGAATTGTGAGCGGATAACAATT
    XbaI
TAATCTAGAAAAGAGGAGAAATTAACCATGAATCCTATAACCGAATTATTAGACGCAACA
                            M  N  P  I  T  E  L  L  D  A  T

TTATGGATCGGCGGAGTTCCGATTCTGTGGCGCGAAATCATCGGCAACGTTTTCGGATTA
 L  W  I  G  G  V  P  I  L  W  R  E  I  I  G  N  V  F  G  L

TTTAGCGCGTGGGCAGGAATGCGACGCATCGTGTGGGCATGGCCCATCGGCATCATAGGC
 F  S  A  W  A  G  M  R  R  I  V  W  A  W  P  I  G  I  I  G

AACGCGCTGCTGTTCACAGTATTTATGGGCGGCCTTTTCCACACTCCACAAAACCTCGAT
 N  A  L  L  F  T  V  F  M  G  G  L  F  H  T  P  Q  N  L  D

CTCTACGGCCAAGCGGGTCGCCAGATCATGTTCATCATCGTCAGTGGTTATGGCTGGTAC
 L  Y  G  Q  A  G  R  Q  I  M  F  I  I  V  S  G  Y  G  W  Y

CAATGGTCGGCCGCAAAACGTCGCGCACTCACCCCAGAAAATGCAGTAGCAGTGGTTCCT
 Q  W  S  A  A  K  R  R  A  L  T  P  E  N  A  V  A  V  V  P

CGCTGGGCAAGCACCAAAGAACGCGCCGGCATTGTGATTGCGGCGGTTGTGGGAACACTC
 R  W  A  S  T  K  E  R  A  G  I  V  I  A  A  V  V  G  T  L

AGCTTTGCCTGGATTTTCCAAGCACTCGGCTCCTGGGGGCCATGGGCCGACGCGTGGATT
 S  F  A  W  I  F  Q  A  L  G  S  W  G  P  W  A  D  A  W  I

TTCGTCGGCTCAATCCTGGCTACCTACGGAATGGCTCGCGGATGGACAGAGTTCTGGCTG
 F  V  G  S  I  L  A  T  Y  G  M  A  R  G  W  T  E  F  W  L

ATCTGGATCGCCGTCGACATAGTTGGCGTTCCTCTACTTTTGACTGCTGGCTACTACCCA
 I  W  I  A  V  D  I  V  G  V  P  L  L  L  T  A  G  Y  Y  P

TCCGCGGTGCTTTACCTGGTGTACGGTGCGTTTGTCAGCTGGGGATTTGTCGTGTGGCTG
 S  A  V  L  Y  L  V  Y  G  A  F  V  S  W  G  F  V  V  W  L

CGGGTGCAAAAAGCAGACAAGGCTCGTGCGCTGGAAGCTCAAGAGTCTGTGACAGTCTGA
 R  V  Q  K  A  D  K  A  R  A  L  E  A  Q  E  S  V  T  V
HindIII
AGCTTCACGCTGCCGCAAGCACTCAGGGCGCAAGGCTGTAGAGAGCCC
```

Figure S8. Nucleotide sequence of the region of pREP-RT containing lacUV5 promoter (in yellow), lac operator (in cyan), and riboflavin transporter gene. The inferred amino acid sequence of the riboflavin transporter is shown in the single letter code. Restriction sites are underlined.

References

1. Bullock, W. O., Fernandez, J.M. and Short J.M. XL1-Blue—a high-efficiency plasmid transforming recA Escherichia coli strain with β-galactosidase selection. Biotechniques 1987, 5, 376-379.

2. Studier, F. W. and Moffatt, B. A. Use of bacteriophage T7 RNA polymerase to direct selective high-level expression of cloned genes. J Mol Biol 1986, 189, 113-130.
